# HOXA11-AS promotes the progression of oral squamous cell carcinoma by targeting the miR-518a-3p/PDK1 axis

**DOI:** 10.1186/s12935-019-0838-6

**Published:** 2019-05-21

**Authors:** Baojun Li, Wei Wang, Susheng Miao, Guofu Li, Yuanjing Lv, Cheng Xiang, Rong Pei

**Affiliations:** 1 Department of Head and Neck Surgery, The Affiliated Tumour Hospital, Harbin Medical University, Harbin, 150081 Heilongjiang People’s Republic of China; 2 Department of Oral Maxillofacial Surgery, The First Affiliated Hospital, Harbin Medical University, Harbin, 150001 Heilongjiang People’s Republic of China; 3 Department of Neurosurgery, The Affiliated Tumour Hospital, Harbin Medical University, No. 150 Haping Road, Nangang District, Harbin, 150081 Heilongjiang People’s Republic of China

**Keywords:** Oral squamous cell carcinoma, HOXA11-AS, miR-518a-3p, PDK1

## Abstract

**Background:**

Long non-coding RNAs (lncRNAs) are promising therapeutic molecules of cancer. Here we aim to study the therapeutic effect and mechanism of a lncRNA, HOXA11-AS, in oral squamous cell carcinoma (OSCC).

**Methods:**

OSCC tissues and adjacent matched paraneoplastic normal tissues used in this study were collected from 42 OSCC patients. The significant downregulation or upregulation of HOXA11-AS expression in OSCC cells was confirmed by quantitative real-time PCR (qRT-PCR). Bioinformatics analysis of StarBase were performed to investigate the potential microRNAs mediated by HOXA11-AS. HOXA11-AS-transfected cells or control cells were subcutaneously injected into nude mice to further determine the effects of HOXA11-AS on OSCC progression in vivo.

**Results:**

qRT-PCR analysis indicated that HOXA11-AS expression was significantly upregulated in OSCC tissues. Functional studies revealed that HOXA11-AS significantly promotes cell proliferation, reduces the percentage of G0/G1 phase cells and enhances the cell invasion in OSCC. Bioinformatics analysis suggested that a microRNA (miRNA), miR-518a-3p, is as a target of HOXA11-AS. Alteration of miR-518a-3p levels by HOXA11-AS transduced to changes in PDK1 expression. In a mouse model of OSCC, HOXA11-AS overexpression promoted tumor growth, concomitant with reduced miR-518a-3p expression and increased PDK1 expression.

**Conclusion:**

Taken together, our study demonstrates that HOXA11-AS/miR-518a-3p/PDK1 axis is an important regulator of OSCC progression and may serve as a potential therapeutic target in OSCC. HARMU20150128, registered at Jan, 28 2018.

## Background

Oral squamous cell carcinoma (OSCC) is a devastating cancer causing 145,000 deaths worldwide as a result, which accounts to 2.1% of the deaths caused by all cancers. It is estimated that there have been over 300,000 new cases in the recent years [1]. Oral cancer consists of a group of neoplasms that affect regions of the oral cavity, pharyngeal regions and salivary glands [2, 3]. In spite of advances in treatment approaches for oral cancer, there has been no improvement in mortality and morbidity in the past 30 years. Percentages of morbidity and mortality in males is at 6.6/100,000 and 3.1/100,000 respectively, whereas for females it is 2.9/100,000 and 1.4/100,000 respectively [4]. The 5-year survival for patients with OSCC varies from 40 to 50%. Diagnosis of OSCC is mostly made in the advanced stages, despite the easy access of oral cavity for clinical examination, largely due to suboptimal efficacy of current diagnosis methods [5].

Long non-coding RNAs (lncRNAs) have gained increasing attention as a new class of regulatory molecules since the completion of the human genome sequencing [6]. A large number of lnRNAs have been associated with cancers. For example, the lncRNA HOTAIR has been shown to have a strong association with poor prognosis and metastasis in gastrointestinal and breast cancers [7, 8]. Its dysregulation has been associated with oral tumorigenesis, with various reports indicating that an increase in HOTAIR expression was associated with stemness and metastasis of OSCC cells [9, 10]. Another lncRNA that has been implicated in OSCC is TUG1 which has been reported to promote OSCC progression by activating Wnt/β-catenin signaling [11]. In another study, a decrease in MEG3 expression has been associated with a poor prognosis in OSCC, and it has been consistently observed that MEG3 inhibited OSCC cell growth and metastasis by suppressing Wnt/β-catenin signaling [12, 13]. An important mechanism of the regulation of lncRNA is through the mediation by miRNAs, which are also key players in cancer [14]. In oral squamous cell carcinoma, a large body of miRNA are uncovered to exert significant regulatory effects, including miR-124 [15], miR-491-5p [16], miR-181a [17], etc., and miRNAs have been utilized as therapeutic targets.

Homeobox A11 antisense (HOXA11-AS), is a newly identified lncRNA, whose gene is located on the HOXA gene cluster. It has been shown to be upregulated in various carcinomas and is generally associated with poor prognosis. Wang et al. [18] found that HOXA11-AS was closely associated with glioma grade and poor prognosis; Richards et al. [19] found a functional variant of HOXA11-AS that inhibited the oncogenic phenotype of epithelial ovarian cancer; Chen et al. [20] found that overexpression of HOXA11-AS in non-small cell lung cancer promoted cell epithelial-mesenchymal transition by repressing miR-200b. Whereas, the biological role of HOXA11-AS in OSCC remains poorly understood.

In the present study, we aim to characterize the role of HOXA11-AS in OSCC. We found that HOXA11-AS expression was significantly up-regulated in OSCC tissues and this correlated with advanced clinical features. We also found that HOXA11-AS significantly promoted cell proliferation and invasion of OSCC cells. The regulation of miR-518a-3p and PDK1 by HOXA11-AS is a mechanism of the tumor-promoting role of HOXA11-AS.

## Materials and methods

### Clinical samples and cell culture

OSCC tissues and adjacent paraneoplastic tissues of 42 pairs used in this study were collected from OSCC patients admitted to The Affiliated Tumour Hospital, Harbin Medical University between July 2015 and April 2017. The cases of OSCC were selected for our study only if clinical data were available, and the patients of OSCC were verified by histopathological analysis of tumor tissue from the surgical resection specimen. None of the patients had received any other treatment, such as radiotherapy or chemotherapy, prior to surgery. Each tissue specimen was stored in liquid nitrogen until use. The corresponding tissues adjacent to tumors were defined as adjacent normal tissues (located > 3 cm away from the tumor) and confirmed pathologically. Characteristics of patients are shown in Table 1. The study protocol was approved by the Ethics Committees of The Affiliated Tumor Hospital, Harbin Medical University with written informed consent obtained from all patients.

**Table 1:** Correlation between lncRNA HOXA11-AS expression and clinicopathological features in OSCC patients

Parameters	Group	N	HOXA11-AS expression		P value
			High	Low	
Age (years)	≤ 60	15	8	7	0.822
	> 60	27	13	14	
Gender	Female	22	12	10	0.442
	Male	20	9	11	
Smoking	Yes	23	13	10	0.168
	No	19	8	11	
Drinking	Yes	16	7	9	0.135
	No	26	14	12	
Position	Tongue	21	11	10	0.086
	Gingiva	13	8	5	
	Buccal mucosa	4	3	1	
	Lip	2	1	1	
Differentiation	Well, moderately	35	22	13	0.012*
	Poorly	7	5	2	
T stage	T1, T2	31	20	11	0.020*
	T3, T4	11	7	4	
N stage	N0	24	17	7	0.001***
	N1–N3	18	12	6	
Clinical stage	I and II	18	11	7	0.039*
	III and IV	24	15	9	

*P < 0.05, ***P < 0.001

The primary normal human oral keratinocytes (NHOK) cell lines and OSCC cell lines, including HN5, CAL-27, Tca8113, SCC-9, SCC-15 and SCC-25 were collected from the Institute of Biochemistry and Cell Biology of the Chinese Academy of Sciences (Shanghai, China). The RPMI-1640 medium (Gibco, Waltham, MA, USA) supplemented with 10% FBS (Gibco), 100 U/mL penicillin and 100 μg/mL streptomycin were used to culture the cells in a humidified atmosphere kept at 37 °C and 5% CO_2_.

### Quantitative reverse-transcription PCR

TRIzol reagent (Invitrogen, CA, USA) was used to extract total RNAs from both tissues and cells. The cDNA synthesis was conducted using PrimeScript RT reagent (Takara, Kusatsu, Japan) and total RNAs. Real-time PCR was performed using an ABI 7900 fast real-time PCR system (ABI, CA, USA) and SYBR Green Master Mix II (Takara). GAPDH and small RNA RNU6B (U6) were used to normalize mRNA and miRNA levels, respectively, and quantified using the 2^−ΔΔCT^ method. Each sample was run in triplicate using samples from independent experiments. The primers used in this study are shown in Table 2.

**Table 2:** Quantitative real-time PCR primers used in this study

Gene name	Primer sequence (5′ to3′)	Amplicon size (bp)
HOXA11-AS	Forward: TTGCCAATCGGGTCACAGCGG Reverse: TCCAGTGCTGGTCTTCGTTGA	278
miR-518a-3p	Forward: CGTCGTTCATGGCGGACTT Reverse: CTTGGGAAAGTGCTTCGTT	193
PDK1	Forward: CTTGAATTCGAGTGCGGAGA Reverse: GCCGGCCAAGACTCGAGTTT	252
GAPDH	Forward: TGAAGGTCGGAGTCAACGGA Reverse: CCTGGAAGATGGTGATGGGAT	225
U6	Forward: CTCGCTTCGGCAGCACA Reverse: AACGCTTCACGAATTTGCGT	257

### Cell transfection

Lipofectamine 2000 (Invitrogen, USA) was used to perform transfections according to the manufacturer’s instructions. miR-518a-3p mimics, miRNA controls, small interfering RNAs (siRNAs) specific to HOXA11-AS and a negative control siRNA were synthesized by GenePharma Technology (Shanghai, China). The HOXA11-AS overexpression plasmid, i.e. pcDNA3.1-HOXA11-AS, was acquired from Genearray Biotechnology (Shanghai, China), which was transfected to SCC-25 cells, followed by G418 selection. Briefly, the SCC-25 cells, which were seeded in 24-well plates at the density of 1 × 10^5^ cells/cm^2^, were grown in complete medium containing 250 mg/mL G418 (Sigma-Aldrich, USA). After 48 h of transfection, cells were cultured for 4 weeks at 1:10 (v/v) in medium with 600 μg/mL G418. The resulting clones, termed as HOXA11-AS cells, were maintained in medium with 300 μg/mL G418. cloning qRT-qPCR was used to confirm HOXA11-AS overexpression in cells.

### Cell proliferation assay

The Cell Counting Kit-8 (CCK8) assay (Dojindo, Tokyo, Japan) was utilized to evaluate cell proliferation. Briefly, cells seeded into 96-well plates (1000 cells/well) were added 10 μL/well of CCK8 for 2 h. Finally, the absorbance at 450 nm was measured using a microplate reader to determine cell viability.

### Cell cycle analysis

The transfected cells were harvested and then fixed with 500 μL of 70% cold ethanol for 2 h. The cells were added with 100 μL of RNase and incubated at 37 °C for 30 min. Then, 400 μL of PI was added, and the cells were incubated at 4 °C for 30 min away from light. The samples were immediately subjected to flow cytometer (FACScan, BD Biosciences). The results were analyzed using CELL Quest 3.0 software.

### Invasion assays

For transwell migration assays, 5 × 10^4^ cells and 1 × 10^5^ cells were seeded in the top chamber of tranwell apparatus (BD Biosciences) with a non-coated membrane and Matrigel coated membrane, respectively. Upper chamber was added serum-free medium and 800 μL of complete medium was added into the lower chambers. After culturing, the cells in the lower chamber were fixed and stained in a dye solution containing 0.1% crystal violet and 20% methanol, followed by imaging with an IX71 inverted microscope (Olympus, Tokyo, Japan). Cell counting was performed using five randomly selected fields.

### Luciferase reporter assay

The wild-type (wt) and corresponding mutant (mut) miR-518a-3p binding sites in the 3′-UTR of HOXA11-AS or PDK-1 mRNA were inserted into the pmirGLO-Report luciferase vector (Genearray Biotechnology, China), yielding HOXA11-AS-wt or HOXA11-AS-mut, and PDK-1-wt or PDK-1-mut. SCC-25 cells were transfected with the reporter plasmid together with either miR-518a-3p mimics and/or pcDNA3.1- HOXA11-AS. After 48 h, a dual-luciferase reporter-assay system (Promega, USA) was used to measure luciferase activity.

### Immunohistochemistry staining

The paraffin embedded human tumor sections were exposed to heat-induced antigen-retrieval in citric acid buffer (pH 7.0), blocked in 5% normal goat serum, and then incubated in 3% hydrogen peroxide. Sections were blocked using 10% normal goat serum and incubated with primary antibodies to PDK1 (1:500; Abcam). Immunodetection was performed using the Envision™ABC kit (GeneTech Co., Ltd., Shanghai, China) according to the manufacturer’s instructions.

### In vivo tumor formation assay

The study was approved by the Ethics Committees of The Affiliated Tumour Hospital, Harbin Medical University. All animal experiments were performed in compliance to institutional guidelines approved by the Use Committee for Animal Care. BALB/c nude mice (female, 4–6 weeks of age) were housed and maintained in a specific pathogen-free room, and were allowed free access to water and food. To initiate OSCC xenografts, 5 × 10^6^ HOXA11-AS-SCC-25 cells (transfected with miR-518a-3p agomir or control agomir) were injected subcutaneously into to the flanks of the nude mice (n = 6 for each group). At 28 days after the initial injection, mice were euthanized in a CO_2_ chamber and tumors were collected. Growth of tumors were measured using calipers every 7 days for 28 days, tumor volumes were calculated using the formula length × width^2^/2.

### TUNEL staining

For TUNEL staining, paraffin embedded mice tumor tissue sections were dewaxed, rehydrated, protease treated, and permeabilized. Then TUNEL staining was performed using an In Situ Cell Death Detection Kit (Roche) according to the manufacturer’s instructions.

### Western blotting analysis

Cell lysis was performed using the RIPA reagent (Beyotime, Shanghai, China) supplemented with 100 μg/mL PMSF (Beyotime, Shanghai, China) and 2 μg/mL aprotinin (Beyotime, Shanghai, China). The proteins in the lysate were separated by 10% SDS–polyacrylamide gel and transferred to a polyvinylidene difluoride (PVDF) membrane. Membranes were then blocked with 5% skim milk powder for 2 h at room temperature, the membranes were then incubated overnight at 4°C using the following specific primary antibodies: PDK-1 (1:1000, Cell Signalling Technology), Cyclin D1 (1:500, Santa Cruz), Cyclin A2 (1:500, Santa Cruz), Caspase-3 (1:1000, CST), MMP-3 (1:500, Santa Cruz), MMP-9 (1:500, Santa Cruz), MMP-13 (1:500, Santa Cruz) and GAPDH (1:1000, CST, 2118). Following this, HRP-conjugated anti-rabbit IgG (1: 2000, CST) was added to the membrane and incubated for 2 h at room temperature. After washing with TBST buffer for three times, bound secondary antibodies were detected by an enhanced chemiluminescence (ECL) system (Pierce Biotechnology, Rockford, USA). GAPDH was used as an internal control.

### Statistical analysis

Data were analyzed using SPSS 19.0 or GraphPad software 7.0. The P-values were analyzed using Student’s t test, one-way ANOVA and Spearman’s test. The correlation analysis was performed by Pearson correlation analysis. P < 0.05 for the difference was considered statistically significant.

## Results

### HOXA11-AS was upregulated in OSCC tissues and cell lines

**Fig. 1:**
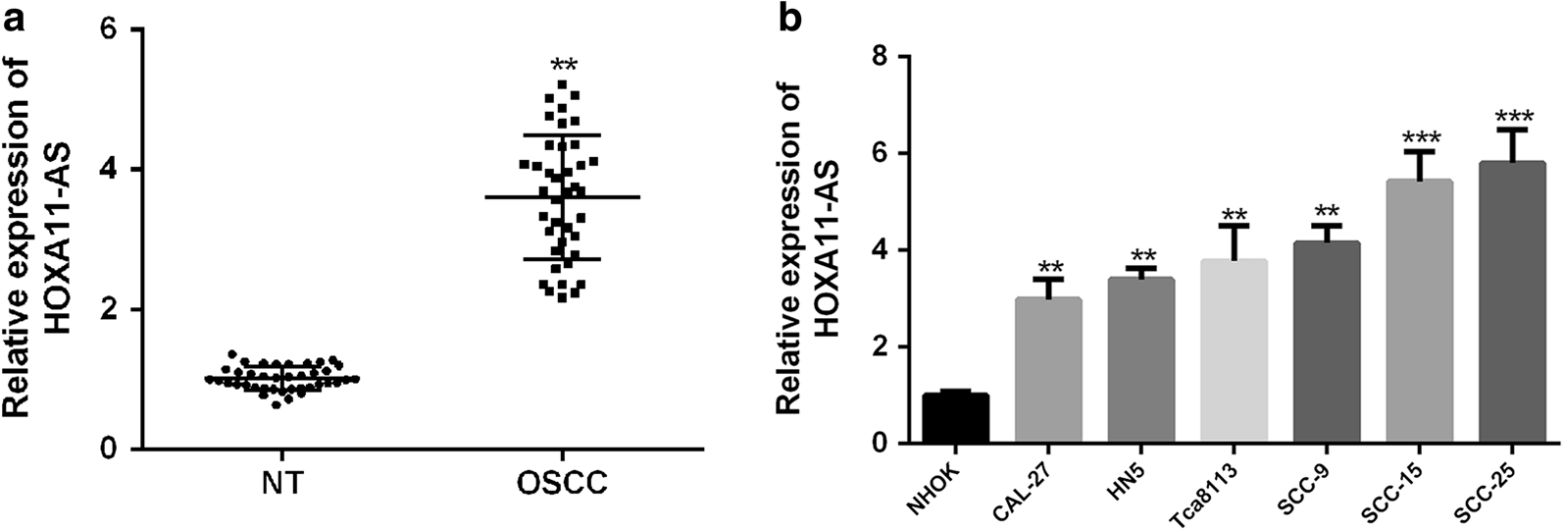
HOXA11-AS is upregulated in OSCC tissues and cell lines. **a** HOXA11-AS expressions in 42 OSCC tissues and paired normal tissues were detected by using qRT-PCR. **b** HOXA11-AS expressions in OSCC cell lines and normal human oral cell line were detected by using qRT-PCR. (**P < 0.01, ***P < 0.001)

To investigate the clinical significance of HOXA11-AS in OSCC, total RNA was first extracted from 42 human OSCC tissues and matched paraneoplastic tissues for qRT-PCR analysis. As showed in Fig. 1a, HOXA11-AS expression in OSCC tissues was markedly higher than that in matched normal tissues (P < 0.01). Next all 42 patients were divided equally into higher and lower HOXA11-AS expression group from the median value (2.99 fold) to evaluate the correction between HOXA11-AS expression and clinic-pathological parameters of OSCC patients. It was shown that HOXA11-AS levels were correlated with grade, clinical stage and lymph node metastasis of OSCC, while HOXA11-AS levels were not correlated to other clinical characteristics, such as gender, age and position in OSCC (Table 2). Moreover, HOXA11-AS expressions in OSCC cell lines (CAL-27, HN5, Tca8113, SCC-9, SCC-15 and SCC-25) were remarkably upregulated compared to that in normal human oral keratinocytes (NHOK) cell lines (Fig. 1b). Taken together, these results indicated that HOXA11-AS might play an oncogenic role in OSCC progression.

### HOXA11-AS enhances the proliferation and invasion of OSCC cells in vitro

**Fig. 2:**
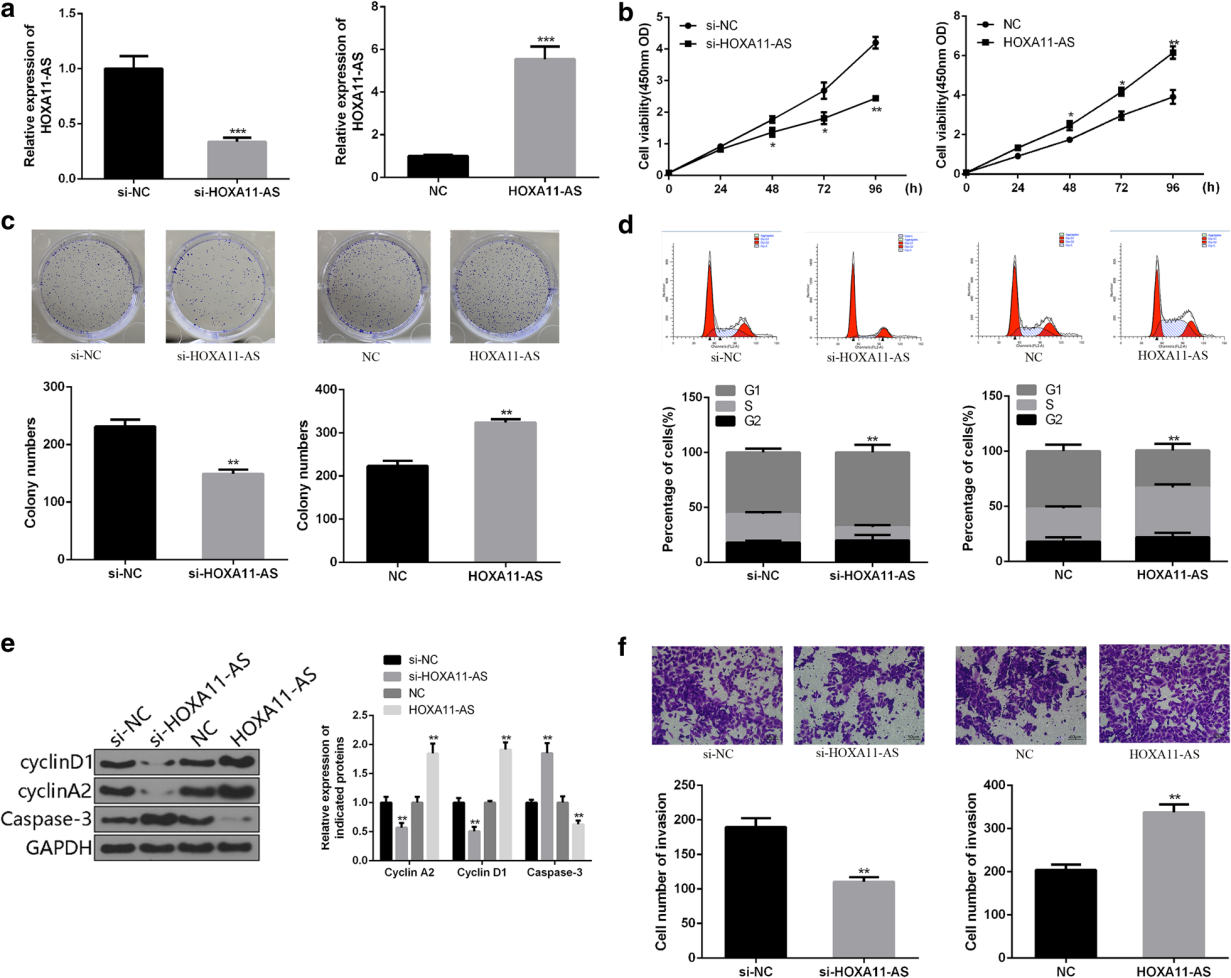
HOXA11-AS enhances the proliferation and invasion in OSCC cells. **a** The transfection efficiency of si-HOXA11-AS or pcDNA3.1-HOXA11-AS in SCC-25 cells was detected by using qRT-PCR. **b** The cell viablity of SCC-25 cells was detected by using CCK-8 assay. **c** The cell proliferation of SCC-25 cells was detected by using colony formation assay. **d** The cell cycle distribution of SCC-25 cells was measured by using flow cytometry. **e** The expressions of cell cycle regulatory proteins and apoptotic marker were measured by western blot. **f** The cell invasion of SCC-25 cells was detected by using transwell assay. (**P < 0.01)

To investigate the functional roles of HOXA11-AS in OSCC cells, the specific siRNA against HOXA11-AS (si-HOXA11-AS) or plasmids (pcDNA3.1-HOXA11-AS) were designed, synthesized and transfected into SCC-25 cells. The significant downregulation or upregulation of HOXA11-AS expression in OSCC cells was confirmed by qRT-PCR (Fig. 2a). The results of CCK-8 assay and colony formation assay indicated that HOXA11-AS downregulation substantially inhibited viability and proliferation of SCC-25 cells. However, upregulation of HOXA11-AS significantly enhanced the cell viability and proliferation of SCC-25 cells (Fig. 2b, c). Consistent result was found in the expression of Caspase-3 (Fig. 2e). The effects of HOXA11-AS on cell cycle arrest was measured by flow cytometry. As shown in Fig. 2d, the transfection of si-HOXA11-AS into SCC-25 cells resulted in a significant increase in the percentage of G0/G1 phase cells, whereas the opposite results was observed in HOXA11-AS-transfected SCC-25 cells (Fig. 2d), accompanied by the changed expression of cell cycle regulatory proteins Cyclin A2 and Cyclin D1 (Fig. 2e). The results of transwell assay showed that cell invasion of OSCC cells transfected with siRNA-HOXA11-AS were significantly lower than control group, whereas cell invasion capacities were significantly promoted by the transfection of pcDNA3.1-HOXA11-AS (Fig. 2f). These data showed that HOXA11-AS enhanced the proliferation and invasion of OSCC cells.

### HOXA11-AS directly targets miR-518a-3p in OSCC cells

**Fig. 3:**
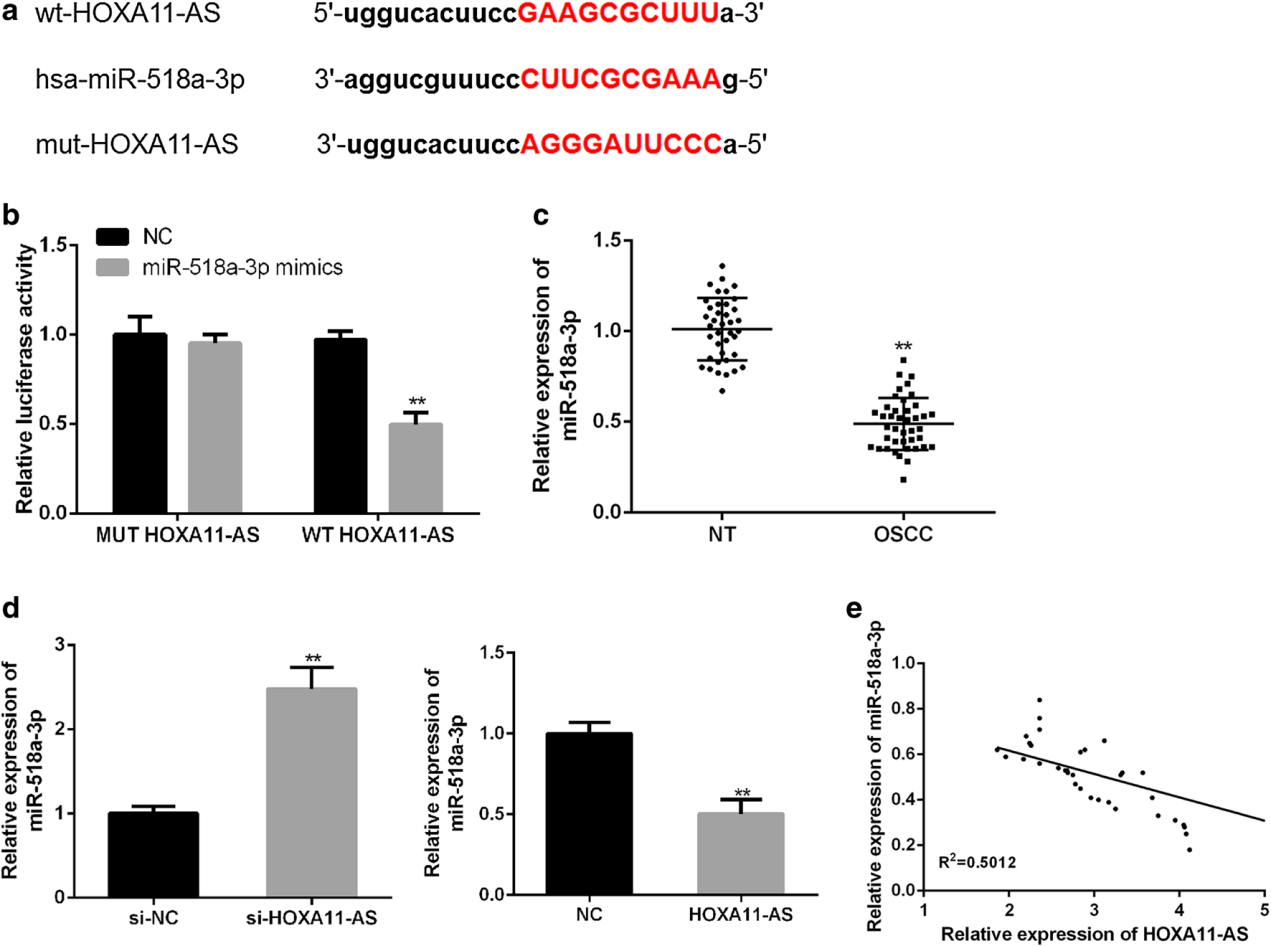
HOXA11-AS directly targets miR-518a-3p in OSCC. **a** Bioinformatics analysis of Starbase showed the predicted binding site between HOXA11-AS and miR-518a-3p. **b** The transfection efficiency of miR-518a-3p mimics in SCC-25 cells was determined by qRT-PCR. **c** WT-HOXA11-AS or MUT-HOXA11-AS was co-transfected into HEK-293T cells with miR-518a-3p mimics or the corresponding negative controls for the detection of luciferase activity. **d** The expression of miR-518a-3p in OSCC tissues and matched normal tissues was detected by using qRT-PCR. **e** The expression of miR-518a-3p was detected in the presence of pcDNA3.1-HOXA11-AS or si-HOXA11-AS by using qRT-PCR. **f** Negative correlation between HOXA11-AS and miR-518a-3p in OSCC tissues. (*P < 0.05, **P < 0.01, ***P < 0.001)

To investigate the potential miRNAs mediated by HOXA11-AS, bioinformatics analysis using StarBase was performed. It was speculated that miR-518a-3p may contain a putative binding site with HOXA11-AS (Fig. 3a). First, the transfection of miR-518a-3p mimics in SCC-25 cells significantly increased the miR-518a-3p expression (Fig. 3b). Moreover, the luciferase reporter plasmids containing wild-type HOXA11-AS and mutant miR-518a-3p binding sites were constructed for a dual-luciferase reporter assay. HEK-293T cells (Thermo Fisher Scientific, USA) were co-transfected with the luciferase reporter plasmid containing the wild type with miR-518a-3p mimics and a decreased reporter activity was observed in Fig. 3c. Moreover, miR-518a-3p expression was significantly lower in OSCC tissues (mean value = 0.48 fold) than that in matched normal tissues (P < 0.01, Fig. 3d). Notably, miR-518a-3p expression was significantly inhibited by the transfection of pcDNA3.1-HOXA11-AS and significantly enhanced by the transfection of si-HOXA11-AS in SCC-25 cells (P < 0.01, Fig. 3e). In addition, a negative correlation between HOXA11-AS and miR-518a-3p (P = 0.024) was confirmed by using correlation analysis in OSCC (Fig. 3f). Taken together, these results indicated that HOXA11-AS may directly targets miR-518a-3p in OSCC.

### HOXA11-AS enhances cell proliferation and invasion by targeting miR-518a-3p in OSCC

**Fig. 4:**
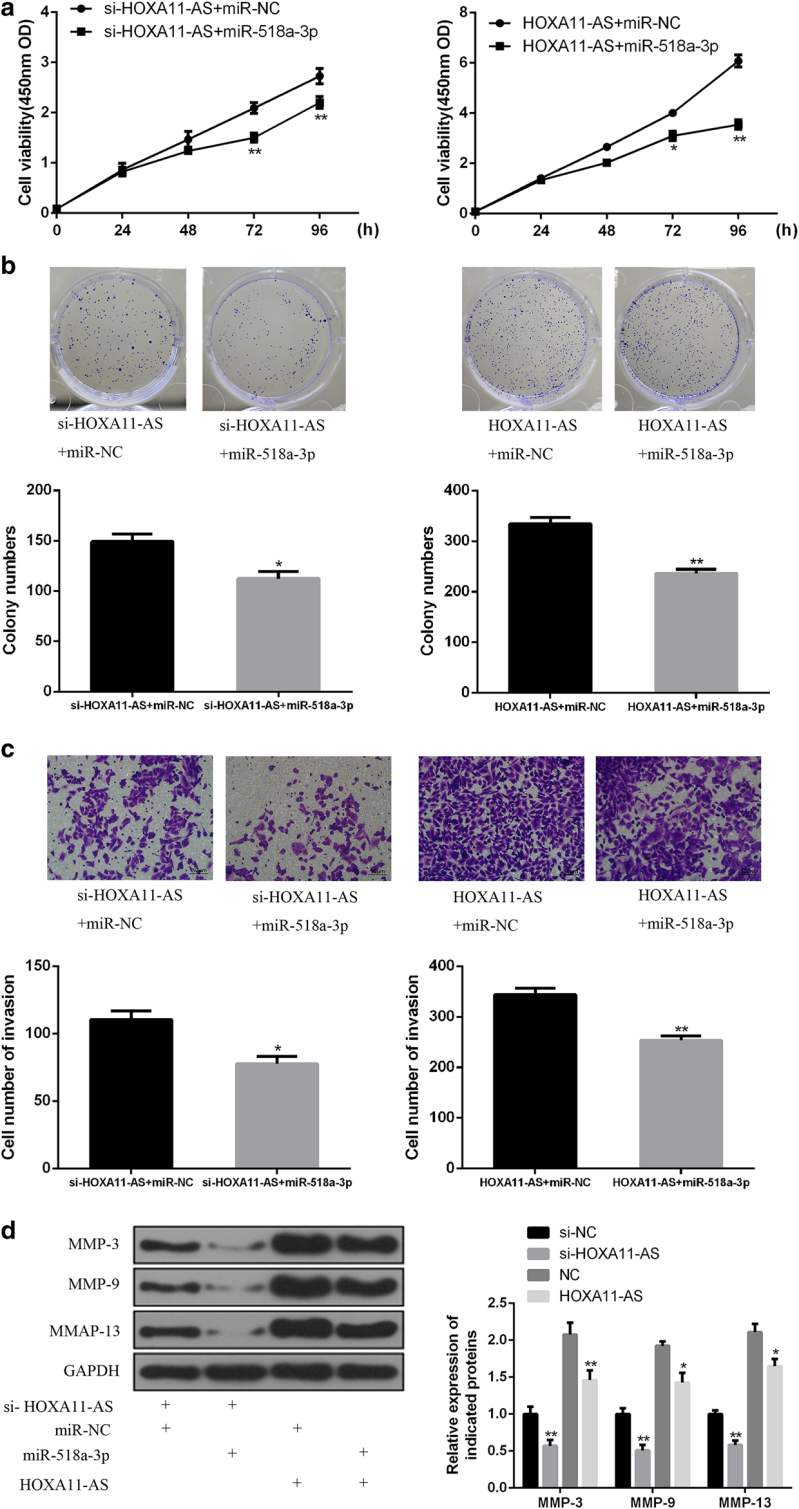
HOXA11-AS promotes cell proliferation and invasion by targeting miR-518a-3p in OSCC. **a**, **b** The effect of miR-518a-3p on cell proliferation in HOXA11-AS-indcued SCC-25 cells was detected by using CCK-8 assay and colony formation assay. **c** The effect of miR-518a-3p on cell invasion in HOXA11-AS-indcued SCC-25 cells was detected by using Transwell assay. **d** The effect of miR-518a-3p on cell invasion marker MMPs was measured by western blot. (*P < 0.05, **P < 0.01)

To further explore whether HOXA11-AS exerts biological functions by targeting miR-518a-3p in OSCC, SCC-25 cells were transfected with miR-518a-3p mimics, simultaneously with pcDNA3.1-HOXA11-AS or si-HOXA11-AS. As showed in Fig. 4a, b, CCK-8 assay and colony formation assay revealed that the enhanced cell proliferation by HOXA11-AS overexpression was prominently reversed by miR-518a-3p mimics, while the inhibited cell proliferation by HOXA11-AS knockdown was further suppressed by the introduction of miR-518a-3p mimics in SCC-25 cells (Fig. 4a, b). Similar trends of transwell assay were observed in Fig. 4c, d, accompanied with the changes of MMP-3, MMP-9 and MMP-13 expressions. Taken together, these evidences suggested that HOXA11-AS promoted cell proliferation and invasion by inhibiting miR-518a-3p in OSCC.

### HOXA11-AS increases PDK1 expression by inhibiting miR-518a-3p

**Fig. 5:**
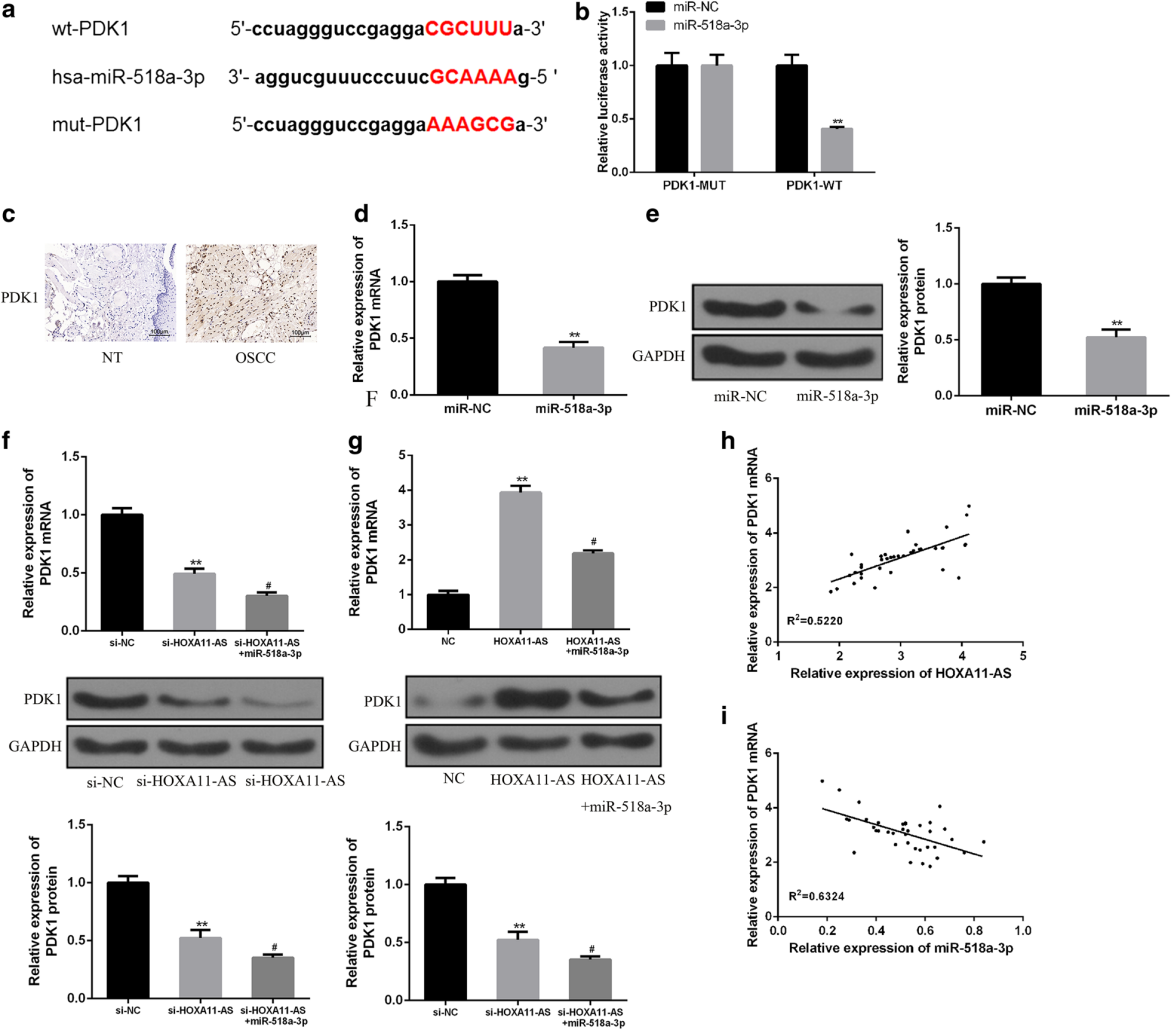
HOXA11-AS increases PDK1 expression by inhibiting miR-518a-3p. **a** Bioinformatics analysis showed the predicted binding site between PDK1 and miR-518a-3p. **b** Luciferase activity was detected in HEK293T cells co-transfected with miR-518a-3p mimics and luciferase reporters containing PDK1 or mutant form. **c** The expression of PDK1 in OSCC tissues or adjacent normal tissues was measured by immunohistochemistry (IHC) assay. **d**, **e** The expression of PDK1 mRNA and protein in indicated groups were detected in SCC-25 cells by using qRT-PCR and western blotting. **f**, **g** The expression of PDK1 protein in indicated groups were detected in SCC-25 cells by using qRT-PCR and western blotting. **h** Positive correlation between HOXA11-AS and PDK1 expression in OSCC tissues. **i** Negative correlation between PDK1 and miR-518a-3p in OSCC tissues. (^#^P < 0.05, **P < 0.01)

To determine the detailed mechanisms of HOXA11-AS and miR-518a-3p in OSCC progression, bioinformatics analysis was used to predict the putative target of miR-518a-3p, and PDK1 was predicted as the downstream target of miR-518a-3p by TargetScan. The binding regions between miR-518a-3p and PDK1 were showed in Fig. 5a. Luciferase reporter assay showed that co-transfection of the luciferase reporter plasmid containing PDK1-WT with miR-518a-3p mimics decreased the reporter activity (Fig. 5b). In the OSCC tissues, PDK1 expression was strongly increased compared to adjacent normal tissues as measured by immunohistochemistry (IHC) assay (Fig. 5c). In addition, PDK1 mRNA and protein expression were also significantly reduced by miR-518a-3p mimics in SCC-25 cells (Fig. 5d, e). These data indicated that PDK1 was a direct target of miR-518a-3p.

Next, we further investigated whether HOXA11-AS promotes OSCC progression by regulating miR-518a-3p/PDK1 axis. As expected, HOXA11-AS knockdown inhibited PDK1 expression, which was further enhanced by miR-518a-3p mimics. Conversely, overexpression of HOXA11-AS significantly enhanced PDK1 expression, whereas miR-518a-3p mimics reversed the HOXA11-AS-induced up-regulation of PDK1 (Fig. 5f, g). Finally, it was demonstrated that PDK1 expression was positively correlated with HOXA11-AS expression (P = 0.014) and negatively correlated with miR-518a-3p expression (P = 0.005) in OSCC tissues (Fig. 5h, i). These results suggested that HOXA11-AS could inhibit miR-518a-3p to promote PDK1 expression in OSCC.

### HOXA11-AS promotes OSCC progression in vivo by regulating the miR-518a-3p/PDK1 axis

**Fig. 6:**
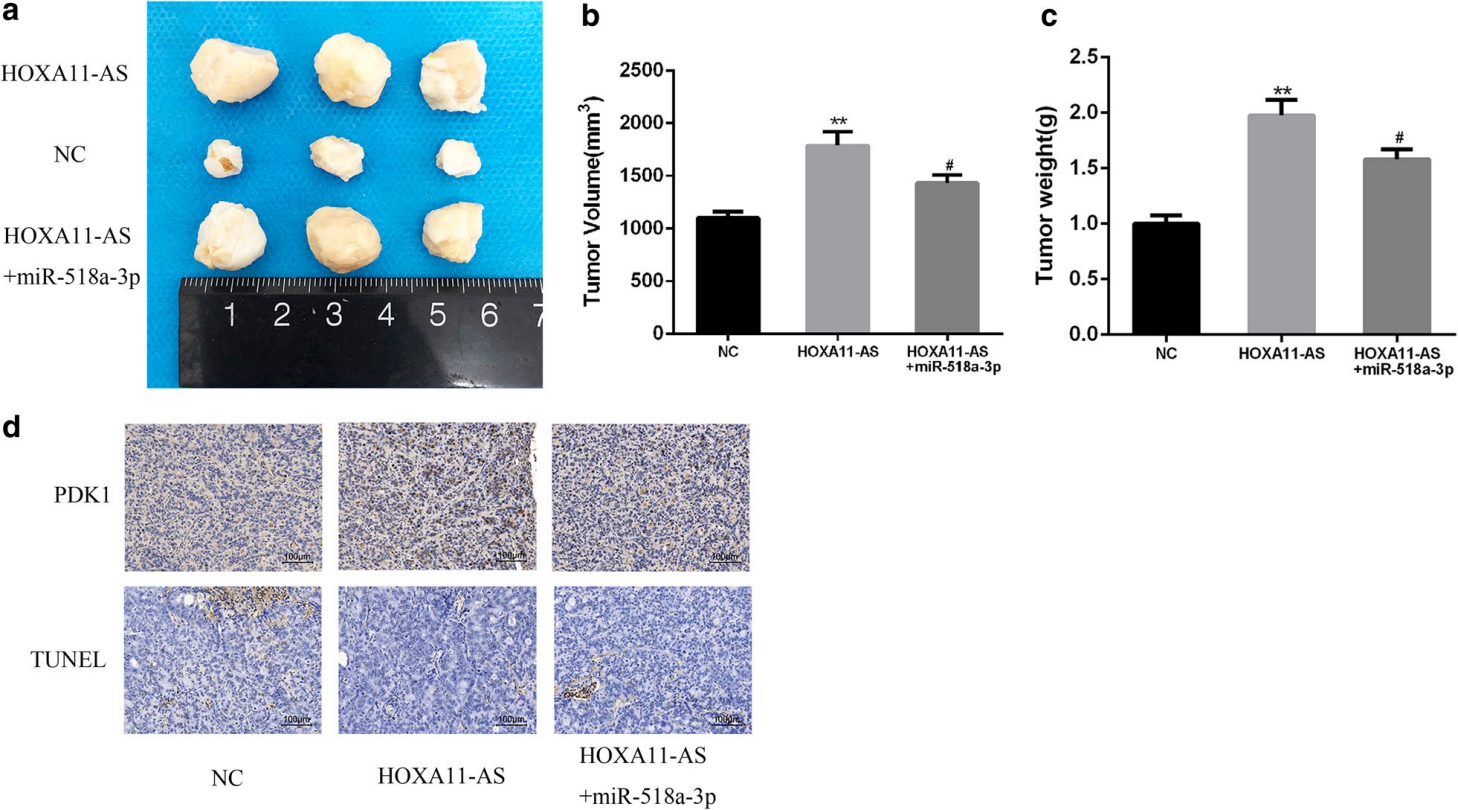
HOXA11-AS promotes OSCC progression in vivo by regulating the miR-518a-3p/PDK1 axis. **a**–**c** The tumor size and tumor weight were directly measured in indicated groups. **d** The expression of PDK1 protein in indicated groups were detected by using IHC and the apoptosis was measured by TUNEL assay (^#^P < 0.05, **P < 0.01)

To further determine the effects of HOXA11-AS on OSCC progression in vivo, HOXA11-AS-transfected cells or control cells (co-transfected with miR-518a-3p agomir or control agomir) were subcutaneously injected into nude mice. As showed in Fig. 6a–c, tumor size and tumor weight were significantly increased in HOXA11-AS group compared to control group. Moreover, miR-518a-3p agomir treatment significantly inhibited tumor growth of HOXA11-AS-transfected cells. In addition, HOXA11-AS overexpression significantly promoted PDK1 expression and reduced the percentage of cells undergoing apoptosis, whereas miR-518a-3p agomir treatment decreased PDK1 expression and increased cell in apoptosis xenograft tumors compared to HOXA11-AS group (Fig. 6d). Taken together, these results demonstrated that HOXA11-AS promotes OSCC progression by regulating miR-518a-3p/PDK1 axis.

## Discussion

We showed that HOXA11-AS was upregulated in OSCC tissues and cell lines. More importantly, HOXA11-AS levels remarkably correlated with differentiation grade, clinical stage and lymph node metastasis of OSCC in patients. It has previously been established in non-small cell lung cancer that high levels of HOXA11-AS expression were correlated with larger tumor size and lymph node metastasis [21]. It has similarly been observed that HOXA11-AS acted as an initiator and facilitator in malignant tumor proliferation and metastasis of gastric cancer. It also acts as a molecular scaffold of LSD1, PRC2 and DNMT1 to epigenetically modify chromosomes in the nucleus, but also occurs as a competing endogenous (ceRNA) that sponges miRNAs in the cytoplasm [22, 23]. We report here for the first time the upregulated expression of HOXA11-AS in OSCC, and HOXA11-AS can be used as a marker for OSCC.

In the current study, we found that HOXA11-AS enhanced the proliferation and invasion of OSCC cells in vitro. Previous studies have shown that HOXA11-AS can be a novel regulator in the proliferation and metastasis of various human cancers. Chen et al. [20] showed that overexpression of HOXA11-AS promoted cell epithelial-mesenchymal transition in non-small cell lung cancer through the repression of miR-200b. Liu et al. [13], while working on gastric cancer, demonstrated the role of HOXA11- AS in promoting cell cycle progression and metastasis. Li et al. [24] implicated the role of HOXA11-AS in promoting breast cancer invasion and metastasis through the regulation of epithelial-mesenchymal transition. Our findings are thus in support of these observations that HOXA11-AS is involved in cell proliferation during cancer.

**Fig. 7:**
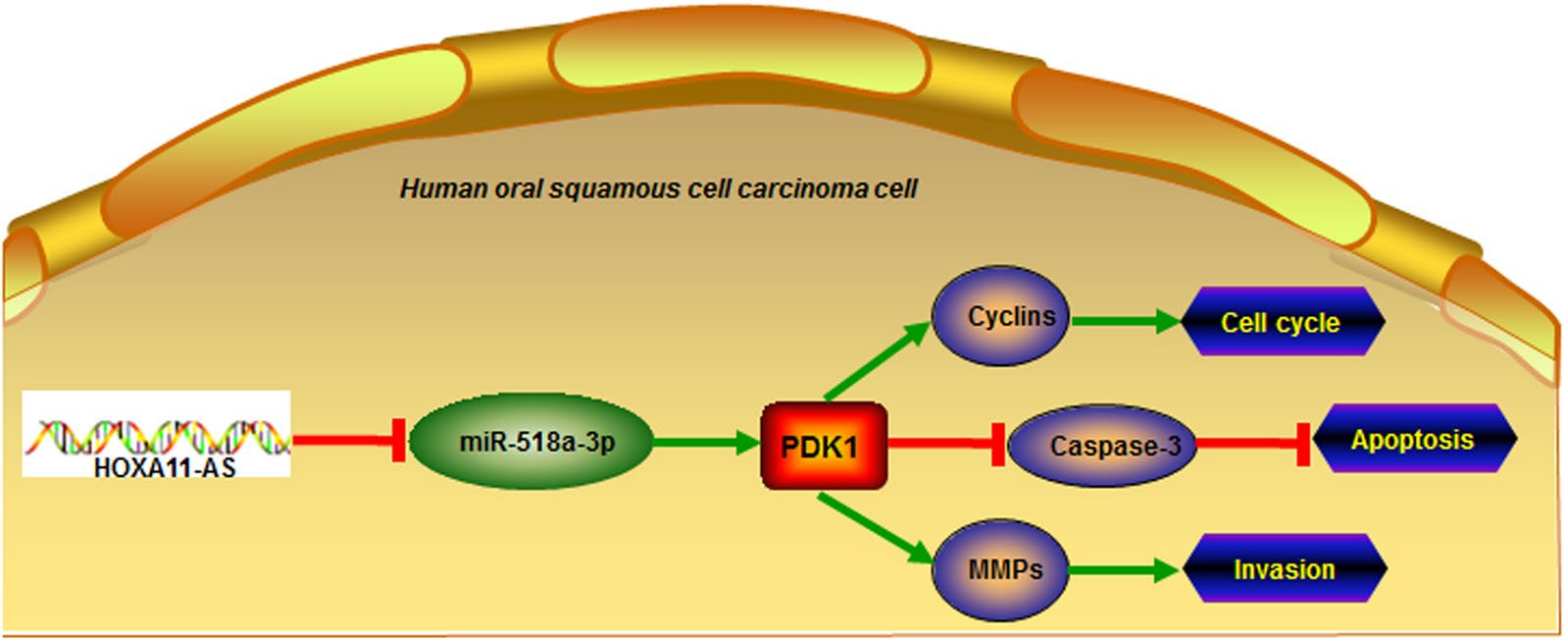
The schematic diagram of HOXA11-AS/miR-518a-3p/PDK1 in OSCC

We sought to explore whether HOXA11-AS exerted its biological functions by targeting miR-518a-3p in OSCC. Our findings suggest that HOXA11-AS promoted cell proliferation and invasion by inhibiting miR-518a-3p in OSCC. A similar observation was made by Qu et al. [25], and they demonstrated that downregulation of miR-518a-3p resulted in the activation of the NIK-dependent NF-κB pathway in colorectal cancer, thereby governing cancer progression. We predicted that the 3-phosphoinositide-dependent protein kinase 1 (PDK1) is the downstream target of miR-518a-3p. Abnormal signaling of the PDK1 pathway has been shown in different cancers. PDK1 is a essential component of PI3K signaling in breast cancer, and PDK1 inhibition can suppress breast cancer progression [26]. PDK1 plays an important role in cell migration regulation, particularly in the context of PTEN deficiency. PDK1 downregulation inhibits migration and metastasis of human breast cancer cells [27]. We further demonstrated that HOXA11-AS knockdown inhibited PDK1 expression, which was further enhanced by transfection of miR-518a-3p mimics. Conversely, HOXA11-AS overexpression significantly enhanced PDK1 expression, whereas transfection of miR-518a-3p mimics reversed the HOXA11-AS-induced upregulation of PDK1, suggesting that HOXA11-AS could inhibit miR-518a-3p to promote PDK1 expression in OSCC. Accumulating evidences demonstrated that PDK1 is a promising therapeutic target and suggested that PDK1 inhibitors may be a valuable tool to prevent cancer progression and metastasis [28]. Our study also underscores the important role of HOXA11-AS/miR-518a-3p/PDK1 axis, in which the transduction of HOXA11-AS1, to miR-518a-3p, and further to PDK1 that governs the expression of cyclins, Caspase-3, and MMPs, contributes to the regulation of OSCC cell cycle, apoptosis and invasion (Fig. 7).

## Conclusion

In conclusion, we showed that OSCC is characterized by HOXA11-AS upregulation, and a remarkable correlation exists between HOXA11-AS levels with differentiation grade, clinical stage and lymph node metastasis of OSCC. The elevated levels of HOXA11-AS contributed to increased proliferation and metastasis of OSCC. The tumor-promoting role of HOXA11-AS is closely related to its regulation of miR-518a-3p and PDK1 axis. The HOXA11-AS/miR-518a-3p/PDK1 axis may serve as a potential therapeutic target in OSCC.

## Abbreviations

OSCC: oral squamous cell carcinoma
lncRNAs: long noncoding RNAs
HOXA11-AS: homeobox A11 antisense
NHOK: normal human oral keratinocytes
CCK8: Cell Counting Kit-8
